# Genome-wide shRNA screen revealed integrated mitogenic signaling between dopamine receptor D2 (DRD2) and epidermal growth factor receptor (EGFR) in glioblastoma

**DOI:** 10.18632/oncotarget.1801

**Published:** 2014-03-07

**Authors:** Jie Li, Shan Zhu, David Kozono, Kimberly Ng, Diahnn Futalan, Ying Shen, Johnny C. Akers, Tyler Steed, Deepa Kushwaha, Michael Schlabach, Bob S. Carter, Chang-Hyuk Kwon, Frank Furnari, Webster Cavenee, Stephen Elledge, Clark C. Chen

**Affiliations:** ^1^ Center for Translational and Applied Neuro-oncology, Division of Neurosurgery, University of California, San Diego, CA; ^2^ Department of Radiation Oncology, Dana-Farber Cancer Institute, Harvard Medical School, Boston, MA; ^3^ Novartis Institutes for BioMedical Research, Cambridge, MA; ^4^ Department of Neurological Surgery and Solid Tumor Program, Ohio State University Medical Center, Columbus, OH; ^5^ Ludwig Institute for Cancer Research, La Jolla, CA; ^6^ Department of Genetics, Harvard Medical School, Boston, MA

**Keywords:** Glioblastoma, DRD2, EGFR, mitogenic signaling

## Abstract

Glioblastoma remains one of the deadliest of human cancers, with most patients succumbing to the disease within two years of diagnosis. The available data suggest that simultaneous inactivation of critical nodes within the glioblastoma molecular circuitry will be required for meaningful clinical efficacy. We conducted parallel genome-wide shRNA screens to identify such nodes and uncovered a number of G-Protein Coupled Receptor (GPCR) neurotransmitter pathways, including the Dopamine Receptor D2 (DRD2) signaling pathway. Supporting the importance of DRD2 in glioblastoma, DRD2 mRNA and protein expression were elevated in clinical glioblastoma specimens relative to matched non-neoplastic cerebrum. Treatment with independent si-/shRNAs against DRD2 or with DRD2 antagonists suppressed the growth of patient-derived glioblastoma lines both *in vitro* and *in vivo*. Importantly, glioblastoma lines derived from independent genetically engineered mouse models (GEMMs) were more sensitive to haloperidol, an FDA approved DRD2 antagonist, than the premalignant astrocyte lines by approximately an order of magnitude. The pro-proliferative effect of DRD2 was, in part, mediated through a GNAI2/Rap1/Ras/ERK signaling axis. Combined inhibition of DRD2 and Epidermal Growth Factor Receptor (EGFR) led to synergistic tumoricidal activity as well as ERK suppression in independent *in vivo* and *in vitro* glioblastoma models. Our results suggest combined EGFR and DRD2 inhibition as a promising strategy for glioblastoma treatment.

## INTRODUCTION

Glioblastoma is the most common form of brain cancer, with approximately 10,000 new cases per year in the U.S.[1, 2].The disease is characterized by invasive growth patterns, rendering complete surgical resection impossible [3]. Moreover, glioblastoma is highly resistant to conventional chemotherapy and radiation therapy [4, 5], with recurrence nearly universal. The median survival of patients afflicted with glioblastoma remains approximately 14 months [2]. Novel therapeutic strategies are desperately needed.

Identification of oncogenic driver proteins responsible for glioblastoma formation and the development of inhibitors against these proteins have led to interests in molecularly targeted therapies [6]. One of the best characterized glioblastoma proto-oncogenes is the Epidermal Growth Factor Receptor (EGFR). While EGFR is frequently mutated or amplified during glioblastoma pathogenesis [7, 8], clinical experiences with EGFR inhibitors have been disappointing [9]. Subsequent studies suggest that glioblastomas possess dynamic molecular circuits, such that EGFR mediated functions can be substituted by activation of alternate pathways [10-16]. In this context, many have proposed that simultaneous inactivation of critical nodes within the molecular circuitry will be required for meaningful clinical efficacy [4, 14].

Here, we conducted parallel genome-wide shRNA screens to identify the critical nodes within the glioblastoma molecular circuitry. We identified a number of G-Protein Coupled Receptor (GPCR) neurotransmitter pathways, including Dopamine Receptor D2 (DRD2). We demonstrate that dopamine antagonists harbor anti-glioblastoma activity that is synergistic when combined with EGFR inhibition. Our data suggest that FDA-approved DRD2 antagonists, such as haloperidol, warrant consideration as potential glioblastoma therapy.

## RESULTS AND DISCUSSION

**Figure 1:**
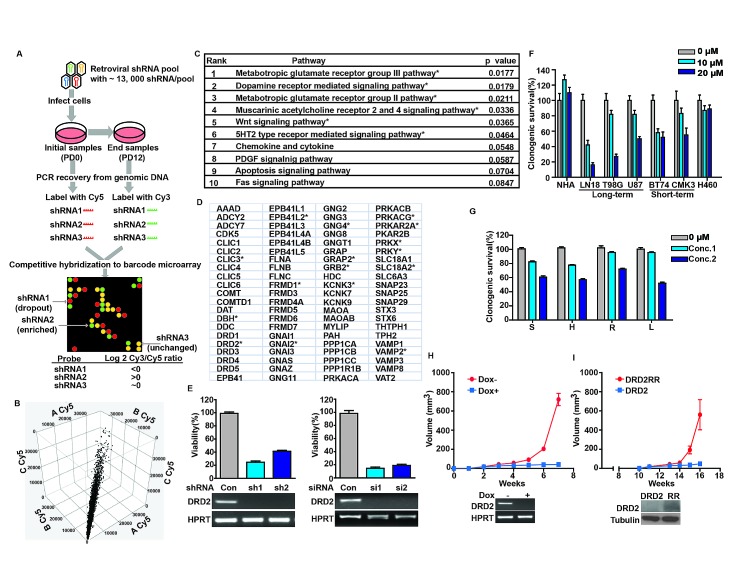
DRD2 is required for glioblastoma growth (A) Schema of genome-wide shRNA screen. Cells were serially passaged after infection with virus containing shRNA constructs. The relative abundance of each shRNA prior to and after passages was subsequently determined (see Methods). (B) Cy5 signal for each gene from three independent U87MG screens (denoted as A, B or C) was plotted against each other (on x-, y-, and z- axes, respectively). High degrees of correlation were seen (R^2^=0.89-0.92). Similar degrees of correlations were seen for Cy3 signals and for other cell lines. (C) PANTHER pathway analysis of pro-proliferative genes for U87MG and A172. * denotes p < 0.05. (D) PANTHER curated DRD2 pathway genes. * denotes pro-proliferative genes identified in our screen. (E) DRD2 silencing by independent sh-/siRNAs compromised clonogenic U87MG growth *in vitro*. Efficiency of DRD2 silencing was determined by RT-PCR (bottom panel). Cells were seeded at ~50% confluency, transfected or infected with siRNA or shRNA constructs for 48 hrs and re-plated in serial dilution for clonogenic survival assessment. (F) Clonogenic growth inhibition of long- and short-term passaged, patient-derived glioblastoma lines by haloperidol. Cells were incubated with haloperidol at the indicated concentrations for the duration of the clonogenic assay. (G) Multiple DRD2 antagonists suppressed U87MG clonogenic growth. S: spiperone (5 μM, 10 μM), H: haloperidol (5 μM, 10 μM), R: risperidone (10 μM, 25 μM), L: L-741,626 (10 μM, 25 μM). Cells were incubated with DRD2 antagonists at the indicated doses for the duration of the clonogenic assay. (H) DRD2 knockdown abolished U87MG subcutaneous xenograft growth. Top: 3×10^5^ cells transduced with inducible shRNA2 against DRD2 were injected subcutaneously into the flanks of Nu/Nu mice. 7 days later, the mice were randomized to drinking water with or without doxycycline (1 mg/ml). Tumor size was monitored every 5-7 days. Bottom: Inducible shRNA silencing of DRD2. U87MG cells with a stably integrated, inducible shRNA construct were treated with doxycycline or vehicle for 72 hrs. RNA was then extracted and DRD2 RT-PCR was performed. (I) Expression of an shRNA resistant form of DRD2 (termed DRD2RR) suppressed the anti-proliferative effects of shDRD2 *in vivo*. Top: U87MG cells transduced with shRNA2 were transfected with DRD2 or DRD2RR, and injected subcutaneously. 7 days post-injection, the mice were fed doxycycline (1 mg/ml) containing water. Tumor size was monitored every 5-7 days. Bottom: DRD2RR expression is resistant to the DRD2 silencing effects of shRNA2. U87MG cells with stably integrated shRNA2 and transfected with DRD2 or DRD2RR (noted here as RR) were treated with doxycycline for 72 hrs. Whole cell lysates were prepared from these cells for DRD2 immuno-blotting.

To identify key nodes required for glioblastoma proliferation, we conducted a genome-wide retroviral shRNA library screen [17, 18] in two glioblastoma cell lines (U87MG and A172) and two lung carcinoma lines (A549 and H460) (Figure 1A). Screen results from independent experiments performed on separate days were highly reproducible (Figure 1B). The targets of the top 1,000 shRNAs that compromised the proliferation of both U87MG and A172 without affecting A549 or H460 growth were analyzed by PANTHER [19]. The analysis revealed a number of pathways previously implicated in regulating cell proliferation and renewal, including PDGF and Wnt [20]. The analysis additionally revealed genes involved in G-protein coupled neurotransmitter receptors (GPCR) signaling (Figure 1C and D). The DRD2 pathway was selected for further characterization because of: i) the availability of FDA-approved DRD2 antagonists, such as haloperidol, that cross the blood-brain barrier [21] and may be repurposed for glioblastoma therapy; ii) findings from previous unbiased chemical screens suggesting that DRD2 antagonists harbor anti-leukemia activity [22], and that such activities may be pertinent to glioblastomas; and iii) previous reports documenting that DRD2 signaling activates MAPK [23-25], and this signaling may contribute to glioblastoma growth [6].

We first validated our screen results using independent sh- and siRNAs against DRD2 (Figure 1E). In all instances, DRD2 silencing compromised U87MG glioblastoma growth by 70-90%. At concentrations that did not impair the growth of Normal Human Astrocytes (NHA) or the H460 lung cancer line, haloperidol caused a 50-70% viability reduction in both long-term passaged glioblastoma lines and glioblastoma stem cell (GSC) lines (Figure 1F). These results were recapitulated using three additional DRD2 antagonists (Figure 1G).

*In vivo*, DRD2 shRNA (shDRD2) expression suppressed U87MG subcutaneous xenograft growth. Mice were implanted with U87MG harboring a doxycycline-inducible shDRD2 construct (dox-shDRD2) and randomized to doxycycline-containing or control water. By week 6, the average xenograft volume in the doxycycline-treated mice was <1% of that in the vehicle-treated mice (Figure 1H). Importantly, expression of an RNAi-Resistant DRD2 construct (DRD2RR) rescued doxycycline-induced the growth-suppressive effect of shDRD2 in this *in vivo* model (Figure 1I). Xenograft formation was noted by week 15 in doxycycline-treated mice that were implanted with U87MG co-expressing dox-shDRD2 and DRD2RR. The growth of these xenografts was slower than that observed for U87MG, suggesting that the phenotypic rescue by DRD2RR was likely incomplete [26, 27]. In contrast, mice harboring U87MG co-expressing dox-shDRD2 and wild-type DRD2 showed minimal tumor growth when fed doxycycline. These results suggest the tumoricidal effect of DRD2 silencing was unlikely the result of off-target effects [28].

**Figure 2:**
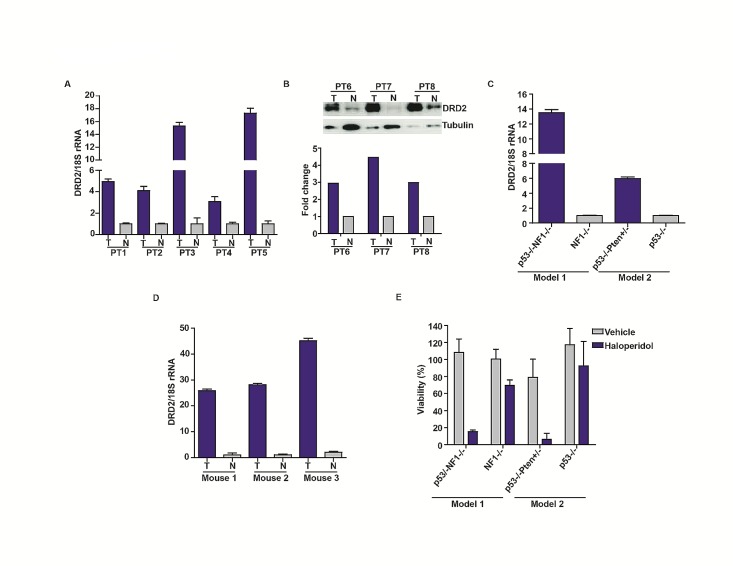
Increased DRD2 expression in glioblastoma specimens (A) Overexpression of DRD2 mRNA in glioblastoma specimens relative to surrounding normal brain tissues. DRD2 mRNA expression was analyzed using qPCR; matched normal-glioblastoma specimens from five patients were tested. T: Tumor; N: Normal brain. (B) DRD2 protein expression was confirmed using three additional matched glioblastoma/normal brain pairs by immuno-blotting. T: Tumor; N: Normal brain. Tubulin: loading control. The ratio of DRD2 to tubulin was quantitated and shown in the bottom panel. (C) Increased expression of DRD2 in GEMM glioblastoma lines. DRD2 mRNA expression was assessed by qPCR. DRD2 mRNA level in a glioblastoma line derived from *hGFAP-Cre+; p53^−/−^*; NF1^flox/flox^ (noted as *p53^−/−^*NF1^−/−^) GEMM [29] was compared to that in an astrocyte line derived from the *hGFAP-Cre+; NF1^flox/flox^ (noted as *NF1−/−*) GEMM. DRD2 mRNA level in a glioblastoma line derived from *hGFAP-Cre+; p53^lox/lox^*; Pten^lox/+^GEMM (noted as *p53 ^−/−^*Pten ^+/−^*) GEMM [30] was compared to that in an astrocyte line derived from an *hGFAP-Cre+; p53^lox/lox^* GEMM (noted as *p53^−/−^*). (D) Elevated DRD2 expression in glioblastomas formed *in vivo* in *Gtv-a Ink4a-Arf−/−* mice stereotactically injected with RCAS-PDGFB-HA [31]. This expression level was compared to the contra-lateral normal cortex. Three sets of matched cortex/glioblastoma specimens are shown. For all qPCRs, the results were normalized to 18S rRNA. Comparable results were obtained when normalized to actin or GAPDH. (E) Sensitivity of GEMM derived glioblastoma and astrocyte lines to haloperidol. Glioblastoma lines were more sensitive to haloperidol relative to astrocyte lines. Cells were seeded at ~50% confluency and treated with 10 μM haloperidol for 5 days. Viability was determined using the CellTiter-Blue viability assay (Promega).

**Figure 3:**
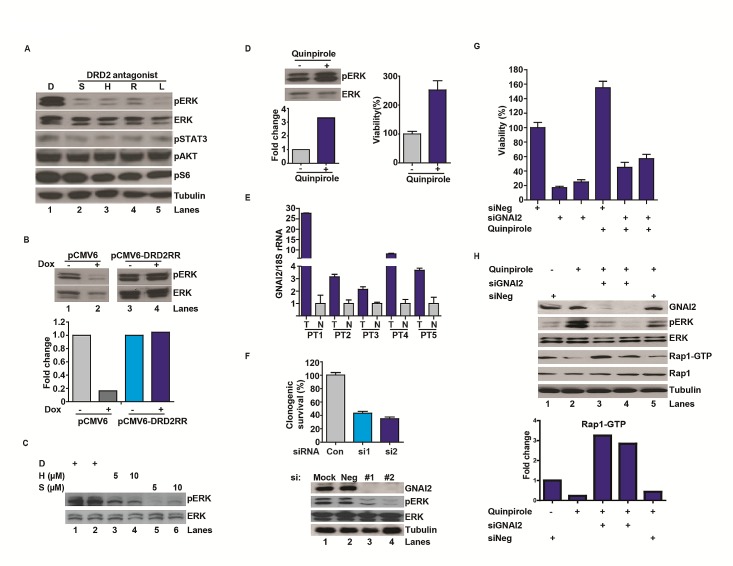
DRD2 signaling through a GNAI2-Rap1-ERK axis (A) DRD2 antagonists decreased pERK accumulation in the U87MG glioblastoma cell line. D: DMSO. S: spiperone (5 μM), H: haloperidol (5 μM), R: risperidone (10 μM), L: L-741,626 (10 μM). Cells were treated with the various antagonists for 1 hr before lysis/immuno-blotting. (B) Decreased pERK activation by DRD2 shRNA can be rescued by expression of an RNAi resistant form of DRD2 (DRD2RR). U87MG lines harboring a doxycycline inducible shDRD2 construct and pCMV6-DRD2RR or pCMV6 were treated with vehicle or doxycycline (1 μg/ml) for 48 hrs before lysis. Bottom panel: quantitative ratio of pERK to ERK. (C) DRD2 antagonists decreased pERK accumulation in the patient-derived CMK3 GSC line in a dose-dependent manner. D: DMSO. S: spiperone. H: haloperidol. CMK3 was treated with the various antagonists for 1 hr before lysis. (D) Treatment with a DRD2 agonist, quinpirole, enhanced pERK accumulation (left upper). CMK3 was treated with quinpirole for 1 hr prior to lysis/immuno-blotting. Lower panel: quantitative ratio of pERK to ERK. Quinpirole promoted cellular growth in the CMK3 GSC line detected by CellTiter-Blue (right). Cells were treated with 10 μM quinpirole for 5 days. (E) qPCR analysis revealed increased GNAI2 mRNA expression in glioblastoma specimens relative to the surrounding normal brain. 5 matched normal-glioblastoma specimens are shown. N: normal brain; T: glioblastoma tumor specimens. GNAI2 mRNA level was normalized to 18S rRNA mRNA. Similar results were obtained when normalized to actin or GAPDH. (F) GNAI2 siRNA transfection compromised proliferation of U87MG cells (top) and reduced pERK accumulation (bottom). #1 and #2 denotes two independent siRNAs against GNAI2. Cells were seeded at ~50% confluency, transfected with siRNAs for 48 hrs, and re-plated in serial dilution for clonogenic survival assessment. (G) The pro-proliferative effect of quinpirole in the patient-derived 1123 GSC line was inhibited by GNAI2 silencing. Cells were transfected with siGNAI2s for 48 hrs prior to treatment with 10 μM quinpirole for 5 days. Viability was determined using CellTiter-Blue (Promega). (H) GNAI2 silencing inhibited quinpirole-induced pERK accumulation and reversed quinpirole-suppression of Rap1-GTP accumulation in U87MG cells. Cells were transfected with siRNAs for 48 hrs prior to the treatment with 10 μM quinpirole for 1 hr.

**Figure 4:**
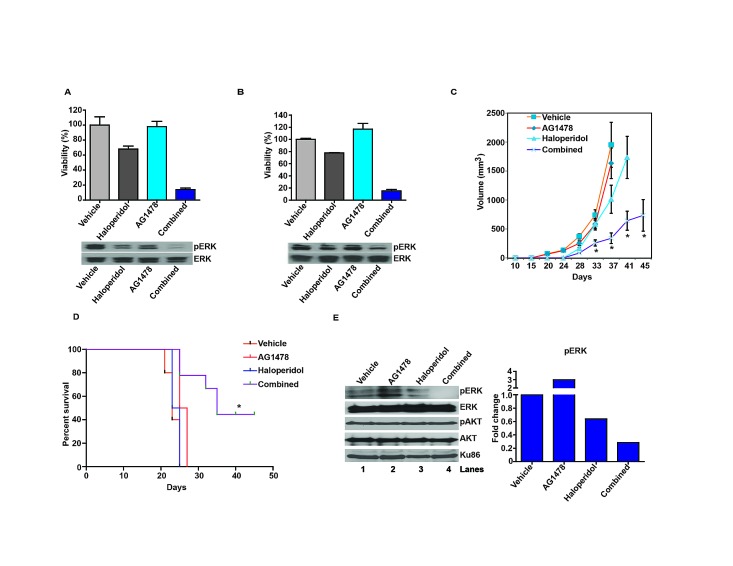
Haloperidol enhances the tumoricidal effect of the EGFR inhibitor, AG1478 (A) Haloperidol enhanced the tumoricidal effect of the EGFR inhibitor, AG1478, against the U87MG glioblastoma line *in vitro* (top). Cells were treated with the various agents for 5 days; viability was determined using CellTiter-Glo (Promega). The enhanced tumor kill correlated with pERK reduction (bottom). For the pERK analysis, cells were treated with the various agents for 1 hr before lysis/immuno-blotting. Combination index (CI) value was 0.32 in U87MG cells using the Chou-Talalay method. (B) Haloperidol enhanced the tumoricidal effect of the EGFR inhibitor, AG1478, against the 1123 GSC line *in vitro* (top). The enhanced tumor kill correlated with pERK reduction (bottom). Cells were treated as described above and viability was measured using CellTiter-Blue (Promega). CI value was 0.29 in 1123 GSCs using the Chou-Talalay method. (C) Combined effect of haloperidol and AG1478 on subcutaneous U87MG xenograft growth. Nu/Nu mice were injected with U87MG cells subcutaneously. 8 days later the mice were randomized to: vehicle (n=7), haloperidol (10 mg/kg, n=8), AG1478 (10 mg/kg, n=8) or combined haloperidol/AG1478 treatment (n=8). *: P<0.05. (D) Combined effect of haloperidol and AG1478 on an orthotopic 1123 mouse model *in vivo*. 7 days after intracranial injection with the 1123 GSCs (1x10^4^ cells), Nu/Nu mice were treated daily by oral gavage with vehicle (n=5), AG1478 (10 mg/kg, n=5), haloperidol (10 mg/kg, n=5) or combined AG1478/haloperidol (n=5). All xenograft experiments were repeated three times. Representative data are shown. *: P<0.05. (E) Synergistic effect of AG1478 and haloperidol in suppressing pERK *in vivo*. 3 weeks after intracranial injection with 1123, Nu/Nu mice were treated with vehicle, AG1478 (10 mg/kg), haloperidol (10 mg/kg), or combined AG1478/haloperidol for 2 hrs, followed by tumor dissection. Whole cell lysates were analyzed for pERK and pAKT levels, with Ku86 as loading control. The quantitative ratios of pERK to ERK are shown in the right panel.

We next determined whether DRD2 was over-expressed in glioblastoma specimens. Relative to tumor-adjacent cerebrum, all glioblastoma specimens showed a 4-17 fold increase in DRD2 mRNA (Figure 2A) or 2-4 fold enhancement in protein expression (Figure 2B). We further tested whether DRD2 expression was associated with any particular molecular subtypes of glioblastoma in The Cancer Genome Atlas (TCGA), but did not identify any specific association (Supplemental Figure 1) [7].

Consistent with observations derived from clinical specimens, DRD2 was highly expressed in GEMM derived glioblastoma lines. DRD2 expression was 14-fold higher in a glioblastoma line derived from an *hGFAP-Cre+*; *p53^−/−^*; *NF1^flox/flox^* GEMM relative to an astrocytic line derived from an isogenic *hGFAP-Cre+*; *NF1^flox/flox^* GEMM [29]. In an independent model, DRD2 expression was 6-fold higher in a glioblastoma neurosphere line derived from an *hGFAP-Cre+*; *p53^lox/lox^*; Pten*^lox/+^* GEMM relative to an astrocytic neurosphere line derived from an isogenic *hGFAP-Cre+*; *p53^lox/lox^* GEMM [30] (Figure 2C). Further, *in vivo* glioblastoma specimens derived from a GEMM where *Gtv-a Ink4a-Arf^−/−^* mice were stereotactically injected with RCAS-PDGFB-HA [31] exhibited 20-40 fold increases in DRD2 expression relative to matched contra-lateral cortex (Figure 2D).

Importantly, the increased DRD2 expression in glioblastomas was accompanied by a dependence on DRD2 for viability. Haloperidol reduced the viability of a glioblastoma line derived from an *hGFAP-Cre+*; *p53^lox/lox^*; Pten*^lox/+^* GEMM by 90%. The same concentration (10 μM) had negligible effects on the growth of an astrocyte line derived from the *hGFAP-Cre+*; *p53^lox/lox^* GEMM (Figure 2E). Similar results were observed in the *NF1 p53* GEMM [29], where haloperidol induced a 20% viability reduction in the astrocyte line derived from an *hGFAP-Cre+*; *NF1^flox/flox^* GEMM and a 80% viability reduction in the glioblastoma line derived from a *hGFAP-Cre+*; *p53^−/−^*; *NF1^flox/flox^* GEMM. These results suggest a therapeutic window for haloperidol in the treatment of glioblastoma.

Previous reports suggest that DRD2 signaling leads to ERK activation [23-25, 32]. We hypothesized that this signaling may contribute to the pro-proliferative effect of DRD2. Supporting this hypothesis, independent DRD2 antagonists suppressed pERK accumulation in U87MG (Figure 3A) by at least an order of magnitude. Suppression of pERK accumulation was also observed after doxycycline-induced DRD2 shRNA knockdown (Figure 3B). Importantly, the suppressive effect of shDRD2 on pERK was abrogated by expressing an RNAi resistant form of DRD2, DRD2RR (Figure 3B). Dose-dependent pERK suppressive effects were similarly observed in a GSC line, CMK3 [33] (Figure 3C). Further supporting this hypothesis, treatment with quinpirole, a DRD2 agonist, induced a 3-fold increase in pERK level (Figure 3D) and a similar increase in the proliferation rate of the CMK3 line (Figure 3D). These results suggest that DRD2 contributes to mitogenic signaling in glioblastomas.

Signaling through DRD2 is tightly coupled to the activation of heterotrimeric G proteins [34-36]. Among these proteins, GNAI2 was previously shown to physically interact with DRD2 [34-36]. GNAI2 was also identified as a pro-proliferative gene in our genome-wide screen (Figure 1C) and was over-expressed in clinical glioblastoma specimens (Figure 3E). Moreover, the expression levels of GNAI2 in TCGA glioblastomas correlated well with those of DRD2 (Supplemental Figure 1). In these contexts, we hypothesized that the proliferative effects of DRD2 signaling may be mediated through GNAI2. Supporting this hypothesis, transfection of independent siRNAs targeting GNAI2 consistently suppressed U87MG growth as well as pERK accumulation (Figure 3F).

If the mitogenic effects of DRD2 were mediated through GNAI2, silencing of GNAI2 should suppress the pro-proliferative effects of DRD2 agonists. Consistent with these predictions, pre-treatment with GNAI2 siRNA (siGNAI2) suppressed the pro-proliferative effects of quinpirole (Figure 3G). However, this suppression was incomplete when compared to the growth inhibitory effects of GNAI2 silencing, indicating that the pro-proliferative effects of quinpirole may be mediated through additional GNAI2-independent mechanisms.

Activation of GNAI2 recruits Rap1 GTPase-activating protein (Rap1GAPII) to decrease the concentration of GTP-bound Rap1 [37]. Since GTP-bound Rap1 antagonizes the function of Ras, an upstream activator of ERK [37, 38], this mechanism may contribute to DRD2-mediated ERK activation. The model predicts that quinpirole treatment should suppress the level of Rap1-GTP (through activation of Rap1GAPII) and induce pERK accumulation. Moreover, these suppressive effects should be reversed by silencing of GNAI2 (which decreases Rap1GAPII activation). Supporting this hypothesis, quinpirole treatment reduced the level of Rap1-GTP by approximately 3-fold (Figure 3H, lanes 5 versus 1). This effect was accompanied by pERK accumulation. The effects of quinpirole on Rap1-GTP suppression and pERK accumulation were abolished when quinpirole was added after pre-treatment with independent siGNAI2s.

Another important pathway that signals to Ras/ERK involves the Epidermal Growth Factor Receptor (EGFR) [7, 39]. Since this axis is frequently hyperactive in glioblastomas [7] and specific EGFR inhibitors have undergone clinical trial [12], we examined whether synergy can be derived by co-treatment with an EGFR inhibitor, AG1478, and haloperidol. Using the Chou-Talalay method [40], we demonstrated *in vitro* synergy between these agents in two glioblastoma models: U87MG and 1123 [41] (Figure 4A and B). Combination index (CI) values were 0.32 in U87MG cells and 0.29 in the 1123 GSC line.

In the subcutaneous U87MG xenograft model, AG1478 or haloperidol, at the concentrations tested, minimally delayed tumor growth. Combined AG1478/haloperidol treatment, however, significantly delayed tumor growth. By post-treatment day 37, the average xenograft volume in the combined treatment group was ~17% of that in the vehicle treated mice (p<0.05). In contrast, the average xenograft volumes of the AG1478- and haloperidol-treated groups were 85% and 49% of the vehicle treated group, respectively (Figure 4C). Importantly, none of the treated mice showed weight loss or other physical manifestations of systemic toxicity relative to the vehicle treated mice.

We next tested the efficacy of the combination in an orthotopic intracranial model. The 1123 xenograft was selected because it better recapitulates the histology, invasiveness, and molecular physiology of clinical glioblastoma than the intracranial U87MG xenograft [41]. In this model, we observed the same pattern of therapeutic response, with only the combined treatment exerting a significant overall survival benefit. All of the vehicle-, AG1478-, or haloperidol- treated mice expired by approximately day 26 post-implantation. In contrast, 50% the combined treatment group survived beyond 45 days (p<0.05) (Figure 4D).

To test the effect of the combined treatment on pERK accumulation *in vivo*, mice were orthotopically implanted with 1123 GSCs and administered vehicle, AG1478, haloperidol, or combined AG1478/haloperidol treatment after xenograft formation. Tumors were extracted 2 hrs after drug infusion and analyzed. These results indicate that the combination of AG1478 and haloperidol synergistically suppressed pERK accumulation *in vivo* (Figure 4E).

In sum, our results showed integrated mitogenic signaling between EGFR and DRD2 in glioblastomas, with conceptual implications for a wider range of cross-signaling between RTKs and neurotransmitter GPCRs [42]. Because antagonists of these GPCRs are clinically available and cross the blood-brain barrier [43], re-purposing of these agents for glioblastoma treatment warrants consideration. As a proof-of-principle, we demonstrated therapeutic synergy between an EGFR inhibitor and a DRD2 antagonist, haloperidol, against glioblastomas using independent models. Interestingly, clinical series have suggested patients undergoing anti-psychotic therapy are less likely to suffer from brain cancer [44]. The clinical familiarity with haloperidol and its FDA approved status should facilitate ease of clinical translation. Moreover, the doses of haloperidol administered in the murine models translate into physiologic serum concentrations achievable in the clinical setting [45]. Nevertheless, it is important to note the superior *in vitro* and *in vivo* effects of DRD2 silencing in glioblastoma ablation relative to haloperidol. This discrepancy suggests DRD2 may contribute to glioblastoma growth through mechanisms unaffected by haloperidol. Insights into these mechanisms should afford novel therapeutic opportunities. Our results further suggest that other FDA approved, blood-brain barrier-penetrating antagonists against GPCR neurotransmitter receptors [34] warrant consideration as potential glioblastoma therapeutic agents.

## METHODS

### Cell lines and Reagents

Human glioblastoma cell lines (U87MG, A172, LN18, T98G) and human lung cancer cell lines (A549, H460) were purchased from ATCC. NHA was purchased from ScienCell. The BT74 GSC line was provided by C. David James (University of California, San Francisco, CA). The 1123 GSC line was provided by Dr. Ichiro Nakano (Ohio State University, Columbus, OH). CMK3 was established as a glioblastoma GSC line derived from a patient specimen and underwent <10 passages before use. Glioblastoma specimens and matched contra-lateral cortex derived from *Gtv-a Ink4a-Arf^−/−^*/RCAS-PDGFB-HA GEMM mice [31] were kindly provided by Dr. Oren Becher (Duke University Medical Center). The *hGFAP-Cre+*; *p53*lox/lox; Pten*^lox/+^* glioblastoma line and *hGFAP-Cre+*: *p53^lox/lox^* astrocyte line were described in [30] and provided by Dr. Frank Furnari (Ludwig Institute for Cancer Research, San Diego). The *hGFAP-Cre+*;*NF1^flox/flox^* astrocyte and *hGFAP-Cre+*; *p53^−/−^*; *NF1^flox/flox^* glioblastoma lines were described in [29] and kindly provided by Dr. Chang-Hyuk Kwon (Ohio State University Medical Center). All adherent glioblastoma lines were grown at 37 °C with 5% CO2, passaged with trypsin and maintained in complete DMEM media (DMEM, 10% FBS (Invitrogen) and 1% penicillin-streptomycin (Gibco)). All GSC lines were maintained in mouse/human NeuroCult medium (Stem Cell Technology) supplemented with EGF, FGF and heparin according to the manufacturer's instructions. NHA cells were passaged using Astrocyte Medium from ScienCell. DRD2 antagonists haloperidol (H), spiperone (S), risperidone (R), and L-741,626 (L), and agonist quinpirole were obtained from Tocris Bioscience. RNAiMax and Lipofectamine 2000 were obtained from Invitrogen. Polybrene was purchased from Sigma.

The experiments involving DRD2 antagonists or sh-/siRNAs were performed in complete DMEM media or in neurosphere media supplemented with EGF, FGF and heparin. DRD2 antagonists were added for 1 hr for the immuno-blotting experiments and for 5-7 days for the viability experiments. For experiments involving siRNAs, cells were transfected with siRNAs for 48 hrs prior to subsequent manipulations. For experiments involving shRNAs, cells were transduced with viral shRNA constructs for 3-5 days prior to subsequent manipulations. For inducible shRNA constructs, transduced cells were treated with or without doxycycline at 1 μg/ml for 5 days before subsequent manipulations.

Experiments involving quinpirole treatment were performed in DMEM with 1% serum or neurosphere media without EGF/FGF supplementation. For viability experiments, quinpirole (10 μM) was added for 5-7 days. For pERK related experiments, lysates were prepared after 1 hr of quinpirole treatment. Cell viability was measured using CellTiter-Glo luminescent cell viability assay kit (Promega) for the adherent lines, CellTiter-Blue cell viability assay (Promega) for the neurosphere cultures, or by clonogenic assays [46].

### Genome-wide shRNA screen

The screen was performed essentially as previously described [17]. In brief, the Hannon-Elledge whole genome pooled shRNA library with 74,705 different MSCV-PM retroviral shRNA constructs targeting human open reading frames was used. The library was split into six pools. For each pool, three replicates of at least 1.3x10^7^ cells of A549, NCI-H460, U87MG, and A172 cells were incubated with an equivalent number of retroviral colony-forming units in media containing 8 μg/ml polybrene (Sigma-Aldrich). Sufficient cells were infected for a 1000-fold representation of each shRNA sequence at a multiplicity-of-infection (MOI) of 1. After selection for stable integrants using 1 μg/ml puromycin, cells were passaged for a total of 12 population doublings (PD), at all times maintaining a minimum of 1.3 x10^7^ cells per replicate. Genomic DNA was extracted from cells harvested both before (PD0) and after the 12 population doublings (PD12). Half-hairpin shRNA-containing sequences were amplified by PCR, purified by agarose gel electrophoresis and labeled with Cy5 (PD0) or Cy3 (PD12). Competitive hybridization to custom Agilent microarrays was performed to determine the relative abundance of each shRNA. The genomic DNA from each triplicate experiment was analyzed on different days. The results of the triplicates were assessed by Pearson correlation analysis.

Scoring was performed as described previously [47]. Briefly, the mean log2 (Cy3/Cy5) ratio was determined for each shRNA sequence using triplicate data. shRNAs with standard deviation of log2 (Cy3/Cy5) ratios greater than the absolute mean log2 (Cy3/Cy5) ratio were excluded from further analyses to reduce false positive hits. For each gene, one point was assigned for each different shRNA sequence for which the mean log2 (Cy3/Cy5) ratio was less than or equal to -1. One half point was assigned for each shRNA sequence for which the mean log2 (Cy3/Cy5) ratio was less than or equal to -0.5. A negative point was assigned for each shRNA sequence for which the mean log2 (Cy3/Cy5) ratio was greater than or equal to +1, and a negative half point was assigned for each shRNA sequence for which the mean log2 (Cy3/Cy5) ratio was greater than or equal to +0.5, to penalize discordant results among shRNA sequences targeting a particular gene. Genes were ranked based on descending total score, with higher rankings given to genes with equivalent total scores but fewer different shRNA sequences targeting the gene. The top 1,000 genes on this list were analyzed by PANTHER pathway analysis (http://www.pantherdb.org/).

### Constructs, Transfection, and Transduction

siRNAs against DRD2 were obtained from Qiagen (DRD2 siRNA #1: GCAACGTGCTGGTGTGCAT; DRD2 siRNA #2: GGGCAGTGCTAGTGAGCTG). GNAI2 siRNAs were obtained from Qiagen (GNAI2 siRNA #1: GGAGCGTATTGCACAGAGT; GNAI2 siRNA #2, GACCATCTGCTTCCCTGAG). Cells were transfected with siRNAs using Lipofectamine according to manufacturer's instructions.

DRD2 shRNAs were provided by the Dana-Farber Cancer Institute Core RNAi facility: DRD2 shRNA #1: GTATCCCTTCTCACAGCACAT; DRD2 shRNA#2: GCCCTTCTTCATCACACACAT. Lentiviral shRNA particle production and infection with a low MOI < 5 were performed according to the manufacturer's instructions and stable cells were generated by selection with puromycin (1 μg/ml) for 5 days prior to further experiments.

For construction of an RNAi resistant form of DRD2 against shRNA#2, a full-length DRD2 clone (pCMV6-DRD2-tGFP) was purchased from OriGene and validated by direct DNA sequencing. The following sequence (G CCC TTC TTC ATC ACA CAC AT) was mutated to (G CCC TTT TTT ATA ACA CAC AT) using the QuickChange II mutagenesis kit (Stratagene) according to manufacturer's instructions. Final constructs were confirmed by sequencing. The pLKO.1-Tet vector (Addgene) was used for inducible shDRD2 experiments.

### RT-PCR and real time Q-PCR

Total RNA was extracted using a previously described Trizol-based method [48] followed by cDNA synthesis using iScript supermix (Bio-Rad). q-PCR was performed and analyzed as previously described [49]. The primers used included: DRD2 forward: 5' GCGGACAGACCCCACTACAA 3' and reverse: 5' AAGGGCACGTAGAAGGAGAC 3'; HPRT forward: 5' CAGGACTGAACGTCTTGCTC 3'; and reverse: 5' CAAATCCAACAAAGTCTGGC 3'.

Real time Q-PCR was conducted using primers: DRD2 Forward: 5' CTCTTCGGACTCAATAACGCA 3' and Reverse: 5' GACGATGGAGGAGTAGACCAC 3'; GNAI2 Forward: 5' TACCGGGCGGTTGTCTACA 3' and Reverse: 5' GGGTCGGCAAAGTCGATCTG 3'; Actin Forward: 5'TGAAGTGTGACGTGGACATC 3' and Reverse: 5'GGAGGAAGCAATGATCT 3'; 18s rRNA Forward: 5'TACCACATCCAAGGAAGGCAGCA 3' and Reverse: 5' TGGAATTACCGCGGCTGCTGGCA3'. GAPDH Forward: 5'ATCATCCCTGCCTCTACTGG 3' and Reverse: 5'GTCAGGTCCACCACTGACAC 3'.

### Immuno-blotting and Rap1 pull down assay

After treatment with DRD2 antagonists or agonist for 1 hr, cells were lysed in RIPA buffer containing 20 mM Tris–HCl (pH 7.4), 150 mM NaCl, 1 mM EDTA, 1% Triton-X 100, 20 mM β-glycerophosphate and 1 mM p-amidinophenyl methanesulfonyl fluoride hydrochloride supplemented with Complete protease inhibitor cocktail (Roche) and PhosSTOP phosphatase inhibitor cocktail (Roche). After sonication in short 1-3 second pulses for 10 times using the 3mm probe set, lysates were cleared by centrifugation at 12,000 rpm for 10 min, and analyzed by immuno-blotting analysis. Antibodies used included: DRD2 (1:1000, Millipore); pERK (1:2000), ERK (1:2000), pAKT473 (1:2000), pS6 (1:2000), and pSTAT3 (1:1000) from Cell Signaling Technology; GNAI2 (1:1000, Proteintech); Tubulin (1:5000, Sigma); Ku86 (1:1000, Santa Cruz Biotechnology).

For the RNAi rescue experiment, U87MG cells transduced with inducible shDRD2 were further transduced with vector or shRNA-resistant DRD2RR followed by appropriate antibiotic selection. Doxycycline (1 μg/ml) treatment was carried out for 48 hrs before cell lysis. For the *in vivo* xenograft experiments, Nu/Nu mice were injected with 1x10^4^ cells. 3 weeks after implantation, mice were treated with vehicle, AG1478 (10 mg/kg), haloperidol (10 mg/kg), or combined AG1478/haloperidol for 2 hrs. The mice were then sacrificed, and the tumors were dissected for lysate preparation as previously described [46].

The Rap1 activation assay was conducted according to the manufacturer's instructions (Thermo Scientific). Briefly, after transfection with siRNA control or GNAI2 siRNAs for 48 hrs, U87MG cells were treated with or without quinpirole (10 μM) for 1 hr. Total cell lysates (500 μg) from each treatment were incubated with agarose beads pre-bound with RalGDS-RBD (Rap1-binding domain) which binds specifically to GTP-bound-Rap1 (incubation for 1 hr at 4°C with gentle rotation). Bound protein was eluted with Laemmli buffer and analyzed by immuno-blotting.

### Tumor and normal human specimens

All research performed was approved by the IRB at the University of California, San Diego Human Research Protections Program and was in accordance with the principles expressed at the Declaration at Helsinki. Each patient was consented by a dedicated clinical research specialist prior to collection. Written consent was obtained for each patient. The consent process was approved by the ethics committee, and all records were documented in our electronic record system. The written consent from patients was also scanned into our electronic filing system. The specimens were collected under IRB 120345X. Pathological analysis of the specimens was performed to verify > 80% tumor cell content by a board certified neuro-pathologist.

### Subcutaneous and orthotopic xenograft models

U87MG cells (3x10^5^ cells in 50 μl for shRNA experiments and 1x10^6^ cells in 50 μl for antagonist experiments) were injected subcutaneously into the flanks of Nu/Nu mice (~7 weeks old) purchased from Charles River Laboratories. The mice were randomized into 4 groups of 7-8 mice: vehicle, haloperidol, AG1478, or combined haloperidol/AG1478 treatment. AG1478 and haloperidol were injected i.p. daily starting 8 days after xenograft implantation. Daily injections took place for 30 days. Tumor volume was calculated based on the formula: volume = (width)^2^ x length/2 every 5-7 days. Mice were sacrificed when they developed tumors measuring greater than 2.0 cm in diameter. Mice were weighed weekly. No significant weight changes were found in the treated versus non-treated groups. For experiments involving doxycycline, doxycycline was dissolved at 1 mg/ml in drinking water with 5% sucrose and given to mice starting 7 days after tumor implantation.

Orthotopic mouse model were conducted in accordance with the ‘National Institutes of Health Guide for the Care and Use of Laboratory Animals’ (NIH publication 80-23). Dissociated GSCs (1x10^4^ cells in 4 μl HBSS) were stereotactically injected into the brains of the nude mice at age 5-6 weeks old. The coordinates were: 1.8 mm to the right of bregma and 3 mm deep from the dura. Starting on day 8, mice received oral administrations of haloperidol (10 mg/kg), AG1478 (10 mg/kg), or both by daily oral gavage. Control mice received 0.5% hydroxypropylmethylcellulose and 0.1% Tween-80 vehicle treatment. Mice with >20% reductions in body weight were sacrificed, and brains were processed for paraffin or frozen section.

### Statistical analyses

Data are presented as means with their respective standard errors (SEM) or as statistical scatter plots generated using GraphPad Prism 5. Student's t test (two-tailed) was used unless otherwise indicated. For survival curves, p values were analyzed using the log-rank (Mantel-Cox) test. P < 0.05 was considered to be statistically significant.

### TCGA glioblastoma transcriptomal analysis

TCGA glioblastoma transcriptomal profiles were downloaded from TCGA Data Portal (http://tcga-data.nci.nih.gov/tcga/). Level 3 normalized, Agilent 244K gene expression mRNA array data was downloaded and gene expression values were median centered. A heat map showing the expression patterns of DRD2, EGFR, and GNAI2 was generated using TIGR Multiexperiment Viewer (http://www.tm4.org/mev.html). Transcriptomal subtypes of glioblastoma were determined as previously described [7].

## SUPPLEMENTARY FIGURE


