# Supplementary material for: Genome-wide shRNA screen revealed integrated mitogenic signaling between dopamine receptor D2 (DRD2) and epidermal growth factor receptor (EGFR) in glioblastoma
Source: Oncotarget. 2014 Mar 7;5(4):882–93. doi: 10.18632/oncotarget.1801 (PMC4011590; doi:10.18632/oncotarget.1801)
Supplement: Supplementary file 1 [file oncotarget-05-882-s001.pdf]

Genome-wide shRNA screen revealed integrated mitogenic signaling between dopamine receptor D2 (DRD2) and epidermal growth factor receptor (EGFR) in glioblastoma

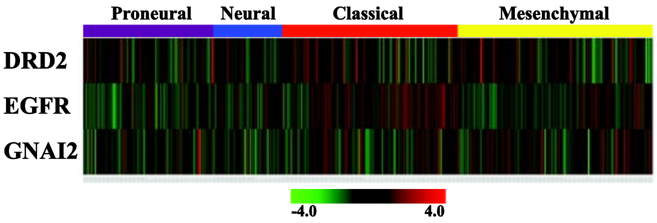

Supplemental Figure 1: Heat map of DRD2, EGFR, and GNAI2 mRNA expression in TCGA glioblastomas.
